# From the Dark Ages

**Published:** 1891-11

**Authors:** 


### FROM THE DARK AGES.

The Earl of Shrewsbury recently purchased the torture implements of the Castle of Nuremburg, and they are now on exhibition in London. The most valuable, as it is the rarest of the whole collection, is the iron maiden (Eiserme Jungfrau). This terror-inspiring torture instrument is made of strong wood, bound together with iron bands. It opens with two doors to allow the prisoner to be placed inside. The entire door is fitted with long, sharp iron spikes, so that when the doors are pressed to, these sharp prongs force their way into various portions of the victim's body. Two entered his eyes, others pierced his back, his chest, and, in fact, impaled him alive in such a manner that he lingered in the most agonizing torture. When death relieved the poor wretch from his agonies—perhaps after days—a trap-door in the base was pulled open and the body was allowed to fall into the moat or river below. Persons were condemned to death by the embraces of the Iron Maiden for plots against the governing powers, parricide, and religious unbelief. The date of this rare specimen is the fifteenth century. A great number of torture machines were apparently constructed with such devilish ingenuity that they would twist and rack the delicate human body to the point of madness, and yet not actually endanger life.

The torture bench, about ten feet long, was used for stretching prisoners, the feet being fastened to one end, the hands to the other, across a roller studded with wooden spikes, called a spiked hare.

The torture chair, the seat being completely covered with sharp wooden spikes ; body, arms, and legs being strapped to the chair, and in some cases two heavy stone weights attached to the feet.

The metal boots, which, being placed on a prisoner's feet, molten lead or boiling oil was poured into them.

A ghastly relic in a black box of coffin-like appearance is the dried head of a child-murderess, still transfixed on a rusty spearhead.

There are tongue-tearers, thumb-screws, mouth-gags, Spanish gaiters for squeezing the legs to pulp, branding-irons, foot-screws, iron-chain gloves to be used when red-hot ; iron nippers, iron-wire whips, heavy stones to be worn round the neck, thief-catchers, and a large number of two-handled executioners' swords. Of the humorous articles—if indeed humor can enter into such grim companionship—we may instance the shame-masks, or brands, worn as signs of degradation for slight offences by men or women, those for the nobility having visors to them like helmets, so that the features were concealed till the penance was over; wooden collars, with bells and tassels, ducking-stools and churn-shaped boxes known as drunkards' cloaks—an uncomfortable garment fitting too tightly round the neck to allow the head to be withdrawn, and, while too heavy to walk about in for any great length of time, yet not quite short enough to permit the wearer to kneel down in them.

Among the pictures is one of Damien, of whom it may be remembered, that, after four horses had failed to pull him asunder, he was afterward tortured with boiling oil, and that not killing, him, was finally bound to a stake and burned to death. Appropriately enough, the instruments are placed in a series of dungeon corridors and cells that the Messrs. Tussaud have erected in the basement of their establishment. One or two cells selected for illustration are copied from the Museum of Antiquities, formerly the Prison of the Inquisition, on Antwerp, and have all the semblance of reality. The first dungeon cell is a small room about 8 feet by 5, so constructed that the inmate died a slow, suffocating death. Perhaps the refinement of cruelty was reached in the plausible escape cell. It was a false beacon to an escaping prisoner, a bait to a poor wretch who, no doubt thought he was about to regain his freedom, may be a connivance bought ; he would hurry down the dark stone corridor to where the dusty gate unlocked or carelessly left ajar would welcome him, to where the bit of shining sky seen through the barred window would gladden his heart, and pushing open the gate with eager hands would at once step into a deep well of water with perpendicular sides.

### BELIEF IN GOD.

This dissatisfaction with the finite, this struggle after the non-finite, this search for an agent for every act, of a mover for every movement, whatever shape it took, whatever name it claimed, forms the primitive and indestructible foundation of man's faith in God. If it is taken away, people may indeed have dogma, and may have creeds, but they cannot have their own ineradicable conviction that there is and that there must be a God. I should go so far as to say that the history of religion is the best proof of religion, just as the growth of the oak tree is the best proof of the oak tree. There may be excrescences, there may be dead leaves, there may be broken branches, but the oak tree is there, once for all, whether in the sacred groves of Germany, or at Dodona, or in the Himalayan forests. It is there, not by our own will, but by itself or by a higher Will. There may be corruption, there may be antiquated formulas, there may be sacred writings flung to the wind, but religion is there, once for all, in all its various representations. You can as little sweep away the oak tree, with all its millions of seeds, from the face of the earth as you can eradicate religion from the human heart. The history of religion teaches us that the one everlasting conviction on which the whole of natural religion has been built from the beginning of the world is true. That is the conviction that there is an Infinite behind the finite, that there is an agent behind all acts, that there is a God in nature.

